# Presence of myocilin sequence variants in Japanese patients with open-angle glaucoma

**Published:** 2008-03-04

**Authors:** MingGe Mengkegale, Nobuo Fuse, Akiko Miyazawa, Kana Takahashi, Motohiko Seimiya, Tomoki Yasui, Makoto Tamai, Toru Nakazawa, Kohji Nishida

**Affiliations:** 1 Department of Ophthalmology, Tohoku University Graduate School of Medicine, Miyagi, Japan; 2 Hiraka General Hospital, Akita, Japan; 3 Yasui Eye Clinic, Miyagi, Japan

## Abstract

**Purpose:**

To examine the myocilin (*MYOC*) gene for mutations in Japanese patients with primary open-angle glaucoma (POAG) and to determine the phenotypes of the patients with the mutations.

**Methods:**

One-hundred thirty-eight unrelated Japanese patients with POAG were studied. Genomic DNA was extracted from leukocytes of peripheral blood, and the three coding exons including the intron-exon boundaries were amplified by polymerase chain reaction (PCR) and directly sequenced bi-directionally.

**Results:**

Two sequence variants were identified, one novel non-synonymous amino acid change (p.Gln297His) and one reported synonymous amino acid change (p.Ala363Thr). These mutations were not detected in the 118 ethnically-matched controls. p.Gln297His was found in a 70-year-old man, who developed POAG at a late age, and his intraocular pressure was high. p.Ala363Thr was found in two cases, and both patients developed POAG at an early age and had high intraocular pressures that responded poorly to medical treatment.

**Conclusions:**

Two non-synonymous variants, p.Gln297His and p.Ala363Thr, indicate that they are involved in the pathogenesis of POAG. p.Ala363Thr has been found in another Japanese population and would be useful in genetic testing.

## Introduction

Primary open-angle glaucoma (POAG) is a genetically heterogeneous disorder characterized by a progressive excavation of the optic disc and a loss of retinal nerve fiber leading to visual field defects. It is most likely a genetically heterogeneous disorder caused by the interaction of multiple genes and environmental factors [1,2]. Adult onset POAG is inherited as a complex trait while juvenile onset open-angle glaucoma (JOAG) has an early onset with high penetrance and an autosomal dominant mode of transmission [3].

To date, at least 14 loci from GLC1A to GLC1N have been linked to POAG, and three genes have been identified, the myocilin (*MYOC*) gene [4,5], the optineurin (*OPTN*) gene [6], and the WD repeat domain 36 (*WDR36*) gene [7]. Mutations of *MYOC* have been found in sporadic and familial cases of POAG worldwide, and their specific phenotype-genotype correlation has been characterized [5,8-24]. Overall, 2%–4% of POAG cases are due to *MYOC* mutations [10,15], although it might be as high as 22.2% [20] to 36% [18] in families with JOAG. *MYOC* encodes 504 amino acid residues [8] and is composed of three exons [25,26]. *MYOC* contains a myosin-like domain and an olfactomedin-like domain, and about 90% of the mutations are clustered in the region coding for the olfactomedin-like domain in exon 3 [5,8,9,11].

The types of mutations found in diverse ethnic populations are different, but the overall incidence of myocilin mutations is similar, which is about 2%–4% in all populations [15]. The most common mutation of MYOC is the p.Gln368Stop, which is found in Caucasians [12] and has not been detected in Japanese. This would suggest that the mutations in MYOC in the Japanese could be different from that of Caucasians and perhaps other ethnic populations.

For the other two glaucoma genes (*OPTN* and *WDR36)*, the results of the screening were difficult to interpret. Thus, the *MYOC* gene is the only glaucoma gene that has been widely evaluated and is accepted to be a causal gene for glaucoma in many populations. The purpose of this study was to examine *MYOC* for mutations in Japanese patients with primary open-angle glaucoma (POAG) and to determine the phenotypes of the patients with these mutations.

## Methods

One-hundred thirty-eight unrelated Japanese patients with POAG (72 men and 66 women; mean age 63.6±14.4 years), who were examined at the Ophthalmic Clinic of the Tohoku University Hospital, Sendai, Japan, were studied. The purpose and procedures were explained to all patients, and an informed consent was obtained. This study was approved by the Tohoku University Institutional Review Board, and the procedures used conformed to the tenets of the Declaration of Helsinki.

Routine ophthalmic examinations were performed on all patients. The criteria used for classifying a patient as having POAG were as follows: 1) applanation intraocular pressure (IOP) greater than 22 mmHg in each eye; 2) glaucomatous cupping in each eye including a cup-to-disc ratio greater than 0.7, 3) visual field defects determined by Goldmann perimetry and/or Humphrey field analyzer consistent with the glaucomatous cupping in at least one eye, and 4) an open anterior chamber angle. The mean IOP at diagnosis was 27.2±5.1 mmHg in the 138 patients with POAG. Patients with secondary glaucoma caused by trauma, uveitis, or steroid-induced were excluded.

Control subjects (62 men and 56 women; mean age 68.0±7.7 years) had the following characteristics: 1) an IOP of less than 22 mmHg, 2) normal optic discs, and 3) no family history of glaucoma. The mean IOP at the initial examination was 14.3±3.4 mmHg in the118 control patients.

Genomic DNA was extracted from peripheral blood leukocytes and purified with the Qiagen QIAamp Blood Kit (Qiagen, Valencia, CA). All three exons that code for a 504 amino acid protein were amplified by olymerase chain reaction (PCR). For the PCR, seven primer sets were used under standard PCR conditions (Table 1). The amplifications were performed at 58 °C annealing temperature. PCR fragments were purified by ExoSAP-IT (USB, Cleveland, OH) and sequenced by the BigDye^TM^ Terminator Cycle Sequencing Ready Reaction Kit (Perkin-Elmer, Foster City, CA) on an automated DNA sequencer (ABI PRISM^TM^ 3100 Genetic Analyzer, Perkin-Elmer).

**Table 1:** Primer sequences used in this study.

**Exon**	**Primer name**	**Primer sequence**
Exon 1	MYOC1–1F	GGCTGGCTCCCCAGTATATA
	MYOC1–1R	CTGCTGAACTCAGAGTCCCC
	MYOC1–2F	AATTGACCTTGGACCAGG
	MYOC1–2R	CTCCAGAACTGACTTGTCTC
Exon 2	MYOC2–1F	ACATAGTCAATCCTTGGGCC
	MYOC2–1R	ATGAATAAAGACCATGTGGG
Exon 3	MYOC3–1F	GGATTAAGTGGTGCTTCG
	MYOC3–1R	AATACGGGAACTGTCCGTGG
	MYOC3–2F	ATACTGCCTAGGCCACTGGA
	MYOC3–2R	CATGCTGCTGTACTTATAGCGG
	MYOC3–3F	GAACTCGAACAAACCTGGGA
	MYOC3–3R	TGAGCATCTCCTTCTGCC

The amplifications were performed at 58 °C annealing temperature. PCR fragments were purified by ExoSAP-IT (USB, Cleveland, OH) and sequenced by the BigDye^TM^ Terminator Cycle Sequencing Ready Reaction Kit (Perkin-Elmer, Foster City, CA) on an automated DNA sequencer (ABI PRISM^TM^ 3100 Genetic Analyzer, Perkin-Elmer).

## Results

Our results showed two *MYOC* variants. The first was a novel *MYOC* variant, a missense mutation at the third nucleotide in codon 297 (CAG>CAC) resulting in an amino acid substitution of glutamine by histidine (p.Gln297His). The second variant was a reported *MYOC* variant, a missense mutation at the first nucleotide of codon 363 (GCT>ACT) resulting in an amino acid substitution of alanine by threonine (p.Ala363Thr) [24]. These mutations were not detected in all normal controls.

### Case 1 (p.Gln297His)

Case 1 was a 71-year-old man diagnosed with POAG at the age of 60 years. He has three brothers and four sisters, but none of them has a glaucoma history. His IOP at the time of diagnosis was 28 mmHg Oculus Dexter (OD), and 45 mmHg Oculus Sinister (OD). He underwent trabeculectomy in his left eye in 2002. In 2007, his best corrected visual acuity (BCVA) in both eyes was 20/40 in the right eye and light perception in the left eye. The optic disc showed characteristic glaucomatous changes with cup/disc ratios of 0.9 in the right eye and 1.0 in the left eye. His IOP was maintained between 20 and 24 mmHg in both eyes under antiglaucoma medication. Global indices of the visual field were a mean deviation (MD) of −7.48 dB by the Humphrey field analyzer in the right eye. The visual fields of the left eye could not be determined.

### Case 2 (p.Ala363Thr)

**Figure 1:**
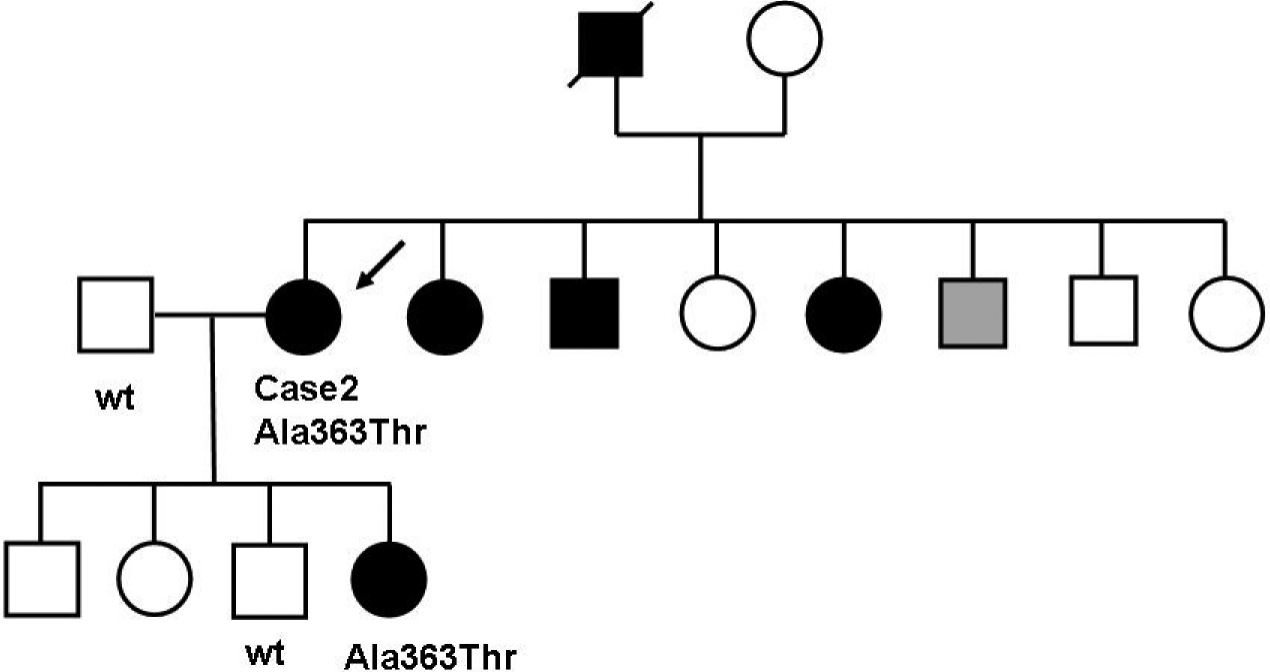
Pedigree of Case 2. Solid squares indicate the affected men, and solid circles indicate the affected women. An arrow points to the proband. The gray square indicates the ocular hypertension and glaucoma suspect. The family included six affected individuals.

Case 2 was a 60-year-old woman who was diagnosed with POAG in 1970 at the age of 24 years (Figure 1). She underwent trabeculectomy four times in her right eye and twice in her left eye from 1980 to 1981. In 1993, the visual acuity of her right eye and left eye was hand motion and counting finger, respectively. The optic discs had the characteristic changes of glaucoma with a cup/disc ratio of 1.0 OD and 0.9 OS. Her IOP was maintained between 15 and 18 mmHg in both eyes with antiglaucoma medication. Her sisters and brother also had glaucoma that was diagnosed before the age of 40 years (Figure 1), but no further medical record and no blood sample was available.

The daughter of the proband was first examined for glaucoma in 1999 at the age of 24. Her BCVA was 30/20 Oculus Uterque (OU). Her IOP were 22 mmHg in both eyes. The optic disc was excavated with the cup/disc ratio of 0.8 in both eyes. Automated static perimetry (Humphrey; program 30–2) revealed a nasal step. Treatment was started with topical antiglaucomatous medications, and her IOP was maintained between 16 and 19 mmHg. Her father and sister were also examined and found not to have any mutations in *MYOC*.

### Case 3 (p.Ala363Thr)

**Figure 2:**
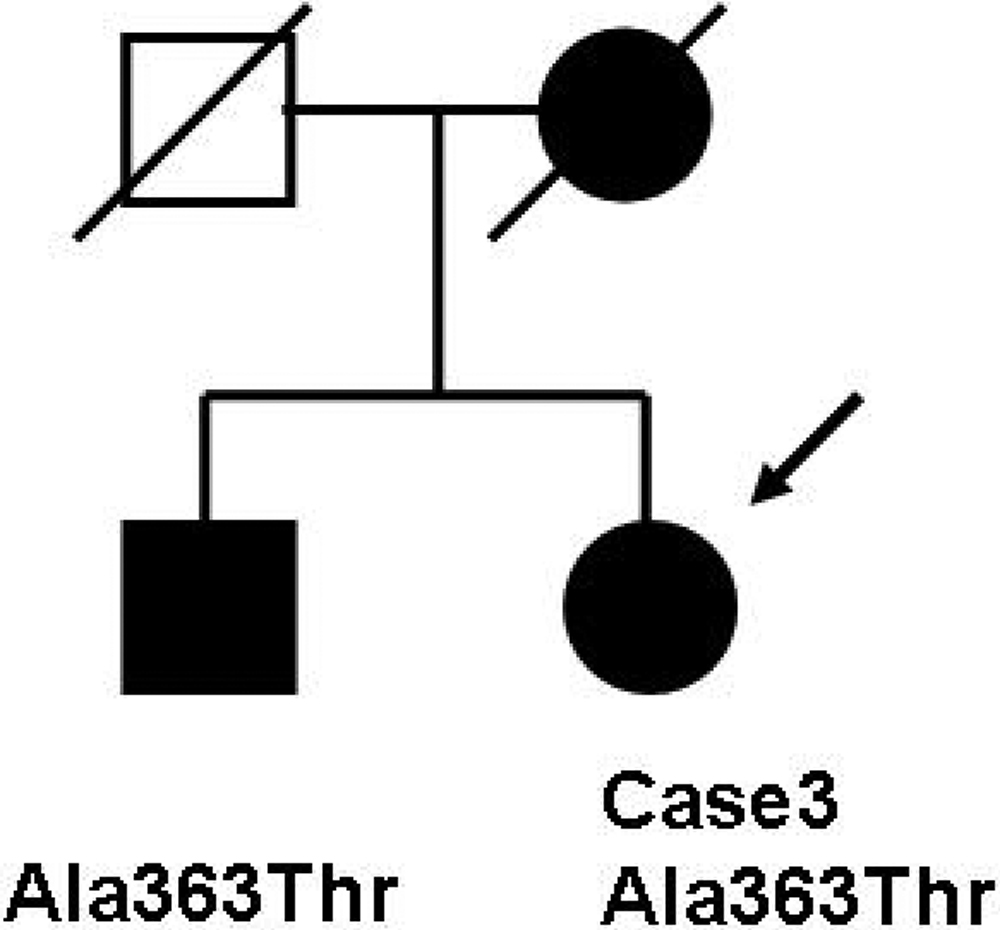
Pedigree of Case 3. Solid circles indicate the proband and affected mother, and the solid square indicates the affected brother. The white square indicates the father, who is an unaffected subject.

This patient was a 65-year-old woman diagnosed with POAG at the age of 44 years. Her brother had also been diagnosed with glaucoma (Figure 2) at the age of 48. As for her mother, no medical record was available regarding the age at diagnosis.

Her IOP at the time of diagnosis was 22–24 mmHg in both eyes. Her BCVA was 20/20 OU. The optic disc showed the characteristic glaucomatous changes in the right eye with a cup/disc ratio of 0.9–1.0, but the ratio was 0.2 in the left eye. Her IOP was maintained around 20 mmHg in both eyes with antiglaucoma medication. She did not undergo glaucoma surgery. In 2007, the optic disc showed optic atrophy with the full cupping in both eyes. Her visual acuity was hand motion in the right eye and 20/200 in the left eye. Goldmann perimetry showed paracentral residual visual fields in the right eye and absolute scotomas in more than one quadrant in the left eye. The proband and her brother were found to have the same p.Arg363Thr mutation.

## Discussion

Our analyses detected two variants of *MYOC* in our Japanese patients with POAG. A novel MYOC variant, p.Gln297His, and a variant, p.Arg363Thr [19,24], which was reported earlier in Japanese patients, were found. The phenotypes of our POAG patients with the two variants were moderate to high IOP, aggressive course without treatment, poor response to topical medication, and moderate response to filtration surgery. The phenotype in these patients is consistent with the phenotype presented for other GLC1A families [3,27-33].

**Figure 3:**
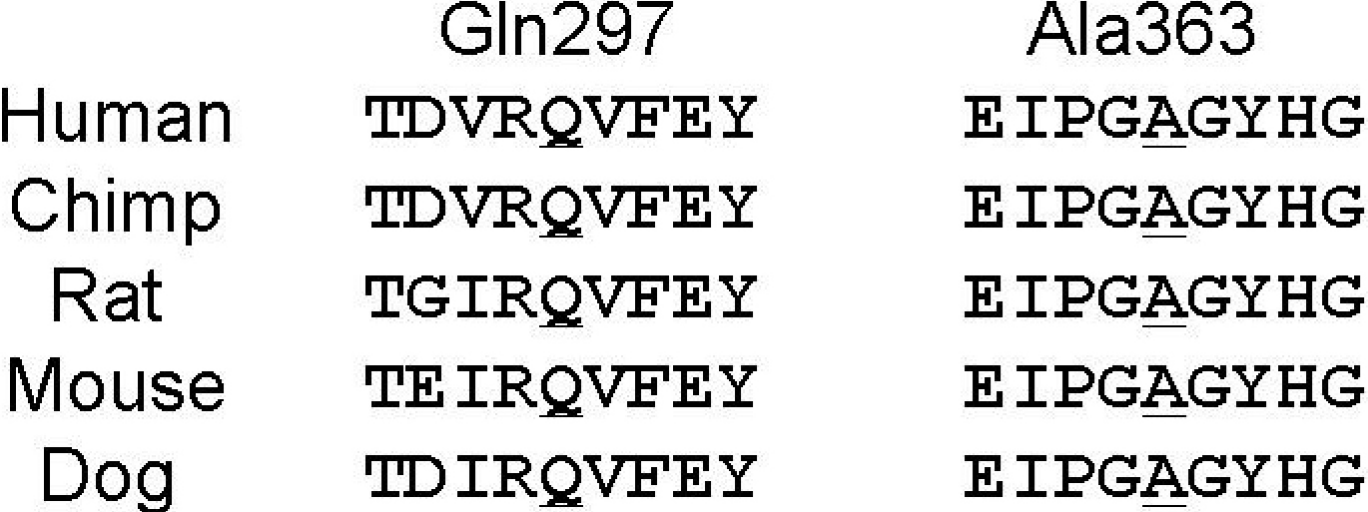
Multiple amino acid alignments and evolutionary conservation of p.Gln297 and p.Arg363 of MYOC variants. Protein domains of MYOC are shown. Gln297 and Arg363 (underlined) are conserved among five species, humans, chimpanzees, rats, mice, and dogs.

In general, Japanese patients with POAG caused by a *MYOC* mutation such as p.Pro370Leu [16] and p.Thr448Pro [17,23] show early-onset at the age 40 years or younger. On the other hand, the POAG caused by a p.Ile360Asn [21] mutation has a late-onset phenotype as it is with the p.Gln368Stop mutation found in Western countries [12,34]. The p.Ala363Thr was segregated as an autosomal dominant mutation (Figure 1 and Figure 2), and *MYOC* orthologs were conserved in humans, chimpanzees, rats, mice, and dogs (Figure 3). p.Gln297His alters the amino acid sequence on the charge (neutral to basic), and p.Ala363Thr alters the amino acid sequence on the polarity (nonpolar to polar). Thus, the conservation of *MYOC* orthologs and changes that alter the charge and polarity can be considered to be probable disease-causing mutations. However, the subject who had the p.Gln297His mutation did not show family history.

**Figure 4:**
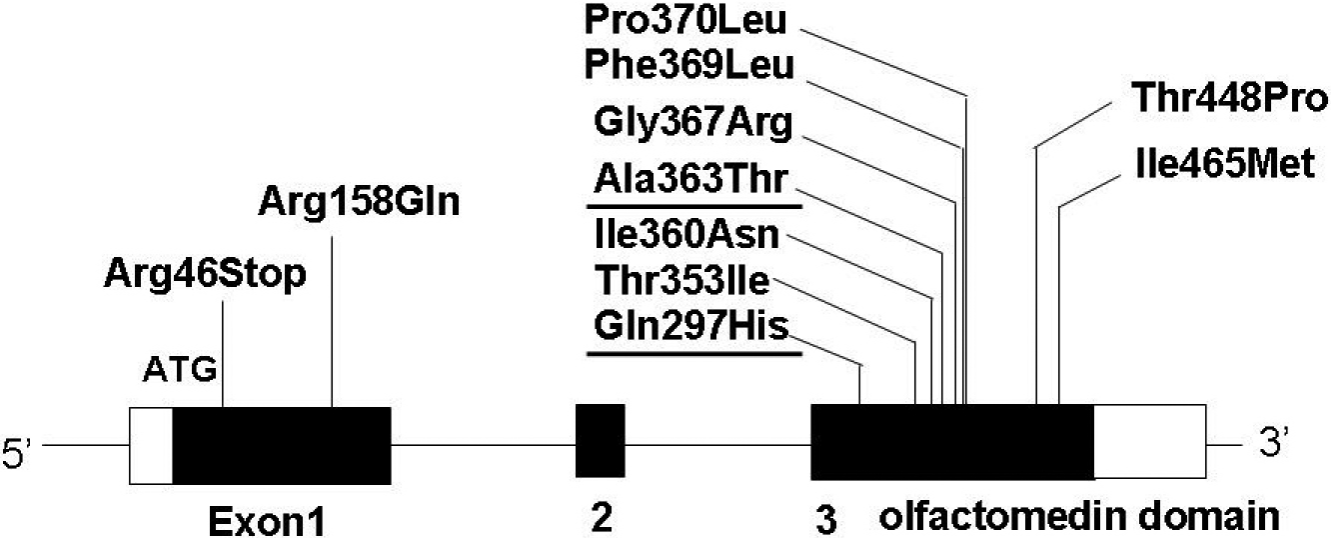
Mutation spectrum found in Japanese individuals. The black boxes indicate the three exons. Nine out of 11 mutations are located in exon 3, the olfactomedin-like domain. The two underlined mutations were found in this study.

About 90% of the mutations are clustered in exon 3 of *MYOC* in different ethnic populations [15], and this incidence is the same in the Japanese population (Figure 4). In this study, *MYOC* mutations were found at the rate of 3/136 (2.2%), which is approximately the same as previous reports on the Japanese and other ethnic populations [15,19]. The p.Ala363Thr mutation was found in another Japanese population and thus may be useful to examine in genetic screening tests [19,24].

Identification of *MYOC* mutations will allow early detection even before the elevation of IOP or the irreversible visual impairment due to damage of the optic nerve. More studies of the function and genotype-phenotype correlation of the *MYOC* gene are required to determine the pathophysiology of POAG.
